# Two Archaeal Metagenome-Assembled Genomes from El Tatio Provide New Insights into the Crenarchaeota Phylum

**DOI:** 10.3390/genes12030391

**Published:** 2021-03-09

**Authors:** Andrés Santos, Pablo Bruna, Jaime Martinez-Urtaza, Francisco Solís, Bernardita Valenzuela, Pedro Zamorano, Leticia Barrientos

**Affiliations:** 1 Laboratory of Molecular Applied Biology, Center of Excellence in Translational Medicine, Universidad de La Frontera, Avenida Alemania 0458, Temuco 4810296, Chile; a.santos01@ufromail.cl (A.S.); p.bruna01@ufromail.cl (P.B.); 2 Scientific and Technological Bioresource Nucleus (BIOREN), Universidad de La Frontera, Temuco 4811230, Chile; 3 Department of Genetics and Microbiology, Faculty of Biosciences, Universitat Autonoma de Barcelona, 08193 Bellaterra, Spain; Jaime.Martinez.Urtaza@uab.cat; 4 Laboratory of Extremophillic Microorganism, Avenida Angamos 601, Universidad de Antofagasta, Antofagasta 1240000, Chile; francisco.solis@uamail.cl (F.S.); Bernardita.valenzuela@uamail.cl (B.V.); pedro.zamorano@uamail.cl (P.Z.); 5 Biomedical Department, Faculty of Health Sciences, Universidad de Antofagasta, Antofagasta 1240000, Chile

**Keywords:** extreme environments, hot-springs, phylogenetic placement

## Abstract

A phylogenomic and functional analysis of the first two Crenarchaeota MAGs belonging to El Tatio geysers fields in Chile is reported. A soil sample contiguous to a geothermal activity exposed lagoon of El Tatio was used for shotgun sequencing. Afterwards, contigs were binned into individual population-specific genomes data. A phylogenetic placement was carried out for both MAG 9-5TAT and MAG 47-5TAT. Then functional comparisons and metabolic reconstruction were carried out. Results showed that both MAG 9-5TAT and MAG 47-5TAT likely represent new species in the genus *Thermoproteus* and the genus *Sulfolobus*, respectively. These findings provide new insights into the phylogenetic and genomic diversity for archaea species that inhabit the El Tatio geysers field and expand the understanding of the Crenarchaeota phylum diversity.

## 1. Introduction

El Tatio (22°20’ S, 68°01’ W) is the largest geyser field in South America, it is located in the Andes mountain at 4300 m above sea level in the Antofagasta Region, Chile. This geothermal field presents several extreme conditions such as high temperatures, high salinity, presence of heavy metals, among the main abiotic factors [1]. These extreme conditions can be observed in soils, hot-springs, sediments, mud pots and many other ecosystems [2] making El Tatio a poly-extreme environment. It is known that bacteria and archaea are capable to colonize diverse habitats including extreme environments and therefore they are considered a reservoir of unique microbial diversity. In this context, studies carried out in El Tatio have reported that microorganism inhabiting this environment live under unique conditions such as the presence of silica, high temperatures (over 86°C), high ultraviolet radiation, low pH and high concentrations of metals and metalloids [3,4]. These extreme conditions have favoured the microorganism evolution and adaptation in El Tatio. Hence, among the microbial adaption mechanism previously described in El Tatio we can highlight metabolisms associated with heat resistance, oxidative stress response, UV radiation resistance, sulphur metabolism, among others [5]. All aforementioned qualities and adaptions of microorganisms inhabiting El Tatio make this environment a unique source for new insights in microbial life, adaptation and microbial diversity. In this regard, the microbial diversity studies in this environment show completely different taxonomic profiles can be found even in close-related sites of El Tatio. Nonetheless, species diversity is often low with only one or a few dominant taxa.

Most microbial diversity studies carried out in El Tatio have been mainly focused on bacteria domain. Thus, the archaea domain remains poorly studied in this environment even when there is evidence about their predominance and functionalities mainly associated to sulphur and methane metabolism. Some of the most abundant archaea inhabiting El Tatio are *Methanococcus*, *Thermoprotei*, *Thermococcus* and Crenarchaeota, where Crenarchaeota has been described as the most predominant [6,7,8]. Crenarchaeota has been characterized as mainly anaerobic, thermophilic and acidophilic phylum for which respiration of elemental sulfur (S°) is common for energy metabolism [9]. Most of the studies where Crenarchaeota phylum has been reported have been accomplished through taxonomic based approaches such as 16S rRNA metabarcoding [10,11]. Hence, since these approaches focus on capturing taxonomic information, the genomic and functional diversity of this archaea phylum inhabiting this environment remains poorly explored. Moreover, to our knowledge, to date there are no Crenarchaeota archaeal genomes reported from El Tatio geysers field. Therefore, in the present study, we characterize the first two archaeal Metagenome Assembled Genomes (MAGs) belonging to the Crenarchaeota phylum from El Tatio Geysers field.

## 2. Materials and Methods

### 2.1. Site Information and Sample Collection

Samples used in this study (Figure S1) were collected from soils contiguous to a geothermal activity exposed lagoon of El Tatio (22°22’00 S 67°59’22.0 W). They were aseptically taken from the upper 5 cm layer of soil and deposited in sterile Falcon tubes, temperature and pH of the sampling point were measured in-situ. Afterwards, samples were kept on ice, transported to the laboratory and frozen at −80°C until DNA extraction. 

### 2.2. DNA Extraction and Sequencing.

Genomic DNA was extracted from soil samples by a modified method using the DNeasy PowerSoil^®^ Kit (QIAGEN, Hilden, Germany). Briefly, samples were heated at 70°C for 10 min and immediately frozen at −80°C for 10 min. Then, enzymatic lysis was carried out with Lysozyme (1:100) and proteinase K (20 mg/mL) (Thermo Fisher Scientific). From this step, the DNA extraction process was carried out according to the DNeasy PowerSoil^®^ Kit (QIAGEN, Hilden, Germany) manufacturer indications. Afterwards, a random amplification protocol was carried out with the GenomiPhi^TM^ DNA Amplification (GE Health Care, Life Sciences, Chicago, IL, USA) to increase the concentration of extracted metagenomic DNA. Finally, DNA quantification was carried out using the One DNA QuantiFluor® ONE dsDNA System (Promega, Madison, WI, USA) on a Quantus fluorimeter. Metagenomic DNA was sequenced under a metagenomic approach on a Novaseq6000 sequencer using a LITE library 150 bp PE at Earlham Institute (Norwich, UK).

### 2.3. Metagenome-Assembled Genomes Binning

Metagenomic reads were quality trimmed using TrimGalore v0.6.0 [12] following default parameters and applying a q28 for quality score, high quality reads were assembled using SPAdes v13.3 [13] with default parameters for the metagenome module (metaspades.py) using a k-mer length of 21, 33, 55, 99 and 127. Contigs longer than 500bp were binned into MAGs using CONCOCT v0.4.0 [14]. Afterwards, in order to improve MAGs quality, the genomic features deviating from the mean coverage, GC and tetranucleotide signature of the MAGs were identified and removed with the outliers module of RefineM v0.0.14 [15] using default parameters. In addition, contigs with incongruent taxonomic classification were removed from each bin using the taxon_profile and taxon_filter methods of RefineM v.0.0.14. Quality and completeness of the archaeal MAGs were assessed using CheckM v1.0.7 [16] and high-quality MAG (>60% completeness, <5% contamination) were selected for downstream analyses. The nucleotide sequences of the MAGs have been deposited into GenBank under the bioproject accession PRJNA695063, Biosamples SAMN18055988 and SAMN18056004.

### 2.4. Taxonomic and Phylogenetic Inference of MAGs

For phylogenetic placement of archaeal MAGs, a taxonomic genome assignment was performed with Centrifuge 10.3 [17] using the NCBI complete genomes bacteria and virus database. Afterwards, a phylogenetic placement was carried out using Phylosift v1.0.1 [18]. For this purpose, our two archaeal MAGs and all representative Crenarchaeota phylum genomes available on the NCBI database (Table S1) were selected. For phylogenomic analysis, archaea domain single-copy genes were identified in our Crenarchaeota genomes collection using the hmm-get-sequences-for-hmm-hits module of anvi’o where 76 archaea single-copy marker genes (Table S4) were obtained and used to build a maximum-likelihood tree using RAxML [19]. A total of 1000 bootstrap replicates were conducted and Anvio7 [20] was used to visualize the phylogenetic tree. Moreover, to obtain a species-level classification an average nucleotide identity (ANI) and average amino acid identity (AAI) were performed using the same Crenarchaeota genomes dataset described for the phylogenetic tree.

### 2.5. Metabolic Reconstruction and Functional Annotation

Gene prediction was carried out with Prodigal v2.6.3 [21] and then annotated with the NCBI’s Cluster of Orthologous Group (COGs) using Diamond v2.0.6 [22]. Metabolic reconstruction of MAGs was performed using the online Rapid Annotation using Subsystem Technology (RAST) [23] using SEED subsystems and the Kyoto Encyclopaedia of Genes and Genomes (KEGG) pathway tool with the Anvi’o7 metabolic reconstruction workflow [20].

### 2.6. Pangenomic Comparison

To characterize and compare functions between our MAGs and their closest neighbors, we identified shared and unique gene clusters and visualized them in Anvi’o using the pangenomic workflow. For this purpose, MAG 9-5TAT was compared to *Thermoproteus uzoniensis* representative genome available on NCBI and MAG 47-5TAT was compared to *Sulfolobus tokodaii* representative genome available on NCBI.

## 3. Results and Discussion

### 3.1. Metagenome-Assembled Genome Binning

The temperature of the sampling site (22°22’00 S 67°59’22.0 W) was 89°C with a pH of 5.4, and it is important to note that the soil sample showed clay characteristics. These temperatures are common for this environment [4,24]; however, our sample possesses a more acidic pH compared with samples from recent studies [8,11] where the pH of samples are in a range of 6 to 8. To obtain the archaeal MAGs, a metagenomic shotgun-sequencing approach was performed. After quality filtering and trimming steps, 57,963,096 high-quality reads were obtained which were assembled resulting in 81,910 contigs. From genome binning, we obtained 20 MAGs belonging to the archaea domain. However, we selected only high-quality MAGs with >60% completeness and <5% contamination. As result, we obtained only two archaeal MAGs (9-5TAT and 47-5TAT). Quality assessment for MAG 47-5TAT showed 67.3% completeness with 1.9% of contamination while the 9-5TAT showed 84.9% completeness and contamination of 2.21% (Table 1).

### 3.2. Phylogenetic Placement of the MAGs

**Figure 1:**
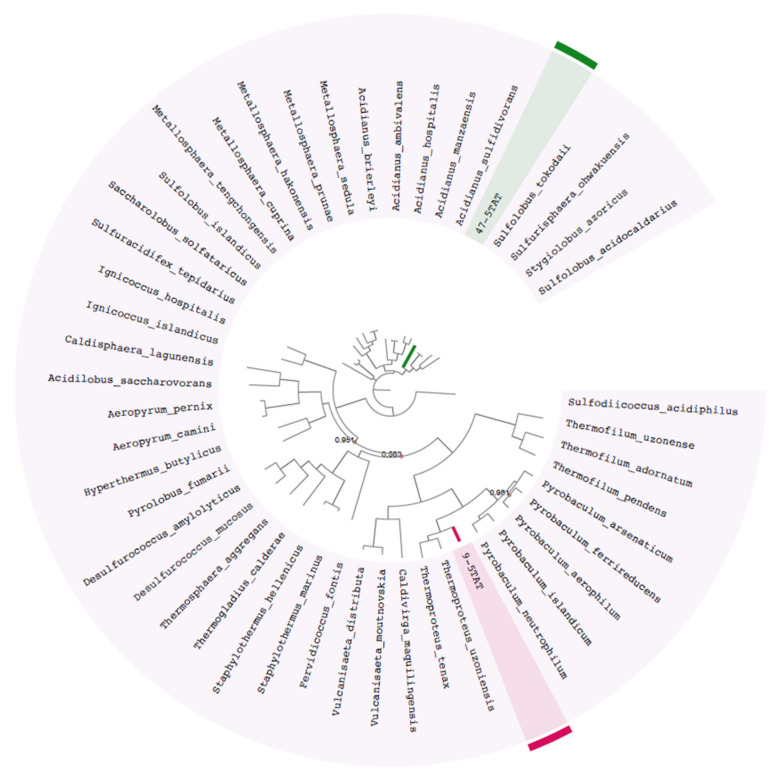
Phylogenetic tree for MAGs. The maximum-likelihood tree was constructed from a concatenated alignment of 76 conserved single-copy marker proteins which were identified in 46 representative genomes of the phylum Crenarchaeota, bootstrap values <100 are displayed on the internal branches. MAG 9-5TAT is highlighted in green and MAG 47-5TAT is highlighted in red.

The first approach used for phylogenetic placement was a taxonomic assignment of the MAG’s contigs using Centrifuge 10.3 [17] where results indicated that both 47-5TAT and 9-5TAT MAGs belongs to the phylum Crenarchaeota of the archaea domain. This approach was unable to give us a lower taxonomic assignment of the MAGs, nevertheless, the second phylogenetic placement approach, determined using 76 single-copy genes showed that MAG 9-5TAT is linked to the genus *Thermoproteus* and MAG 47-5TAT is affiliated to the *Sulfolobus* genus (Figure 1). Additionally, we determined that *T. uzoniensis* is the closest neighbour for MAG 9-5TAT and *S. tokodaii* is the closest neighbour for MAG 47-5TAT.

Crenarchaeota have been described as an archaeal phylum that dominates hot springs environments. This phylum is characterized by their ability to tolerate extremely high temperature and acid conditions [25]. Moreover, Crenarchaeotes are mainly isolated from hot-springs and soils containing high sulphur and sulphides concentrations [26]. Regarding archaeal detected in El Tatio, Crenarchaeota is one of the most abundant phyla which is evidence of a significant role in this environment [8,11,27]. In addition, Crenarchaeota phylum has been described as one of the main active archaeal phyla in other Chilean extreme environments such as Huasco Salar [28,29]. Even though Crenarchaeota have been reported in El Tatio by other authors; based on our bibliographic review there are no previous reports of Crenarchaeota strains isolated from El Tatio geysers field.

Since preliminary analyses for phylogenetic placement were not able to provide a species-level identification, MAGs were compared by ANI to all the representative Crenarchaeota phylum genomes available in NCBI database. Results indicated that the closest neighbour for 9-5TAT was *T. uzoniensis* with an ANI of 76.9% (Table 2). Since an ANI >97% is considered the species cut-off and an ANI >80% is considered for closely related species [30] our results indicate that MAG 9-5TAT may be a species close to the *Thermoproteus* genera. On the other hand, MAG 47-5TAT was phylogenetically close to *S. tokodaii*; however, obtained ANI was 58.7% (Table 2), this ANI value is not resolutive enough to link our MAG at the species level, however it could be phylogenetically associated to the *Sulfolobus* genus. Moreover, is important to note that ANI values <80% are considered not trusted [30]. Therefore, these ANI values only indicate that our MAGs are different to all representative Crenarchaeota genomes available in the NCBI database and, therefore, a species-level phylogenetic placement could not be possible. Thus, we carried out a genus-level placement using AAI analysis and results indicated an AAI of 70.35% for 9-5TAT where *T. uzoniensis* was confirmed as the closest species. On the other hand, for MAG 47-5TAT a 66.02% AAI with *S. tokodaii* confirmed this strain as the closest neighbour. A 70% AAI is considered as a genus-level cut-off [31] therefore our results show that MAGs 9-5TAT and 47-5TAT likely represent a novel species in the genus *Thermoproteus* and probably a new genus in the phylum Crenarchaeota which is closely related to the *Sulfolobus* genus, respectively.

### 3.3. Functional Annotation and Metabolic Reconstruction of the MAGs

Functional annotation of MAG 9-5TAT showed a genome length of 1,447,267. Overall, 2109 ORFs were predicted and 827 COGs were detected. Metabolic reconstruction indicates that MAG 9-5TAT possess energy metabolism associated with carbon fixation, metabolism of methane, nitrogen and sulphur. Most Crenarchaeota are anaerobic and elemental sulphur (S°) respiration is common; however, many other electron acceptors are used by various species and studies have already reported and demonstrated that Crenarchaeota plays essential roles in sulphate reduction [32,33]. All metabolic predicted pathways suggest that MAG 9-5TAT probably grows chemolitho-autotrophically with S° as an energy source and CO_2_ as the main carbon source. These energy metabolisms are very similar to the described for representative species of the genus *Thermoproteus* such as *Thermoproteus tenax*, *Thermoproteus neutrophilus* and *T. uzoniensis* [34].

Additionally, several resistance mechanisms to toxic compounds were detected in MAG 9-5TAT, most relevant are cooper, cobalt, zinc, cadmium and mercury resistance pathways. Metabolic machinery for the response to oxidative stress and carbon starvation was also predicted. All aforementioned is completely related to the environmental conditions of El Tatio, where oxidative stress and the presence of heavy metals are a common characteristics [1]. However, a microorganism capable of thriving under this type of stress are capable of withstanding and converting toxic metals into harmless forms and they are relevant nowadays in a biotechnological context because they represent an efficient means of environmental decontamination [35].

Functional annotation of MAG 47-5TAT showed a genome length of 1,265,490 bp. Overall, 746 COGs were detected from 1673 predicted ORFs. Metabolic reconstruction indicates that MAG 47-5TAT possess the capacity of carbon fixation and it uses methane and sulphur for energy metabolism. Carbon fixation and sulphur metabolism have been described as core functionalities in the *Sulfolobus* genus [36]. All the aforementioned suggest that this MAG could be also chemolithoautotrophic which uses S° as energy source and CO_2_ as the main carbon source. This MAG showed methanogen capability, in this regard, among methanogenic archaea previously detected in the El Tatio, genera *Methanospirilum* and *Methanobrevibacter* have been reported as the most prevalent [27]. In addition, they identified a group of unclassified Crenarchaeota which were associated with this functionality. Identification of the Crenarchaeota group was based on the use of a metabarcoding approach, therefore our results corroborated the findings and provided new insights into the methanogen archaea living in El Tatio geysers field.

Central carbon metabolism of MAG 47-5TAT showed a metabolic potential to degrade glucose, galactose, and also possesses metabolic pathways to degrade polysaccharides such as glycogen. The aforementioned has been reported as a common characteristic for *Sulfolobus* species such as *Sulfolobus solfataricus* [37,38]

Nitrate/nitrite ammonification and ammonia assimilation pathways were identified in this MAG. This metabolism is interesting for MAG 47-5TAT since only a few *Sulfolobus* species have gained the ability to utilize additional nitrogen sources such as nitrate and *S. tokodaii* is one of them [25,39]. Therefore, this is a relevant shared metabolism between MAG 47-5TAT and *Sulfolobus* genus and probably is the distinctive functionality linking MAG 47-5TAT with this *Sulfolobus* species. In addition, several oxidative stress pathways such as protection from reactive oxygen species, CoA disulfide thiol-disulfide redox system and rubrerythrin were also identified. Some Crenarchaeotas are considered a source of metabolites of biotechnological interest. Within this context, archaea capable of inhabiting environment with high temperatures and acidic pH are expected to produce temperature and acid-stable enzymes, biomaterials and metabolites [36]. In consequence, considering all the metabolic potential of MAG 47-5TAT it could represent a novel source of these type of metabolites. 

### 3.4. Pangenomic Analysis of the MAGs

**Figure 2:**
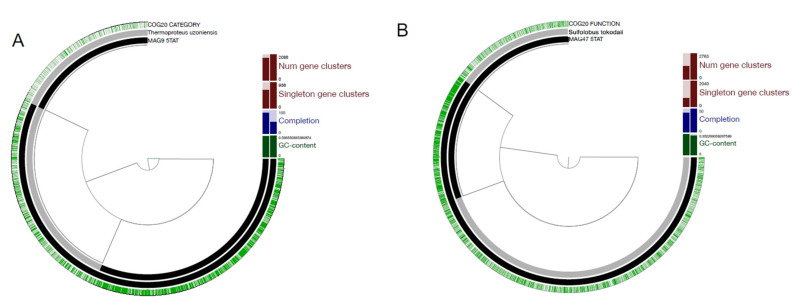
Pangenome analysis of MAG 9-5TAT (**A**) and MAG 47-5TAT (**B**) compared with their closest species produced in Anvi’o (Eren et al., 2015).

Finally, because the two MAGs that have been analysed in this work likely represent new species in the phylum Crenarchaeota, we studied the metabolic differences between both MAG 9-5TAT and 47-5TAT and their respective closest neighbours (Figure 2). 

When analysing the genome metabolic profiles in the genomic reconstruction, six metabolic pathways not detected in the closest species were found in MAG 9-5TAT, which are related to energy metabolism (Table S3). Among the Crenarchaeota phylum, the key differential elements are related to the energy metabolism and in many cases, these differences determine the genus or the species in the classification of an archaea of this phylum [9,25,40]. Based on our results, this feature could be the most relevant difference and key element for this MAG to be classified as a new species within the genus *Thermoproteus*. Similar findings were obtained through the pangenomic functional analysis for MAG 47-5TAT. Results showed that, compared with its closest phylogenetic neighbour, MAG 47-5TAT possess eight metabolic pathways (Table S2) not detected in the closest species, related to central carbon metabolism and energy metabolism. In consequence, the singular features that make this MAG a potentially new species in the phylum Crenarchaeota are more numerous than the ones observed in MAG 9-5TAT. These findings were also supported by the ANI and AAI value. For both MAG 9-5TAT and 47-5TAT, the unique genomic and metabolic attributes are probably the results of the selective pressures that an extreme environment such as El Tatio exert on microbial communities, allowing unique metabolic adaptations and the speciation of archaea living in this ecosystem. Therefore, adding more efforts for the study of archaeal species in this ecosystem is extremely relevant to identify novel species that can expand the archaea domain and also could give new insight in the tree of life.

## 4. Conclusions

In summary, both MAG 9-5TAT and MAG 47-5TAT likely represent new species in the genus *Thermoproteus* and the genus *Sulfolobus*, respectively. These findings provide new insights into the phylogenetic and genomic diversity for archaea species that inhabit the El Tatio geysers field and expand the understanding of the Crenarchaeota phylum diversity.

## Figures and Tables

**Table 1:** Archaeal MAGs used in the present study. CDSs: coding sequences; COGs: orthologous groups of proteins.

MAG	Completeness (%)	Contamination (%)	GC Content	Genome Length	CDSs	COGs	KEEG Pathways
9-5TAT	84.9	2.21	57.5	1,447,267	2109	827	121
47-5TAT	67.3	1.9	35.2	1,265,490	1673	746	71

**Table 2:** Average nucleotide identity (ANI) and Average amino acid identities (AAI) for MAGs.

MAG	Closest Species	Orthologous Genes	Mean AAI	Mean ANI	Closest species GC	Closest Species Genome Length
9-5TAT	*T. uzoniensis*	848	70.35	76.9	59.6	2,839,046
47-5TAT	*S. tokodaii*	1093	66.02	58.7	35.2	1,960,328

## Data Availability

This study generated sequences of two new archaea genomes. This data can be found at https://www.ncbi.nlm.nih.gov (accessed on 31 January 2021) under the accession number provided for each nucleotide sequences.

## Supplementary Materials

The following are available online at https://www.mdpi.com/2073-4425/12/3/391/s1, Figure S1: Sampling site in El Tatio geysers field, Table S1: Crenarchaeota genomes used for phylogenetic placement, Table S2: Metabolisms detected for MAG 47-5TAT which were not present in *Sulfolobus tokodaii*, Table S3: Metabolisms detected for MAG 9-5TAT which were not present in *Thermoproteus uzoniensis*, Table S4: List of single-copy genes used for MAGs phylogenetic placement.
