# Supplementary material for: Two Archaeal Metagenome-Assembled Genomes from El Tatio Provide New Insights into the Crenarchaeota Phylum
Source: Genes (Basel). 2021 Mar 9;12(3):391. doi: 10.3390/genes12030391 (PMC7999037; doi:10.3390/genes12030391)
Supplement: Supplementary file 1 [file genes-12-00391-s001.zip › Supplementary/Table S4.docx]

Supplementary table 4. List of single-copy genes used for MAGs phylogentic placement.

| ATP-synt_D |
| --- |
| ATP-synt_F |
| Adenylsucc_synt |
| AdoHcyase |
| Archease |
| CTP-dep_RFKase |
| CarS-like |
| DNA_primase_lrg |
| DUF357 |
| DUF359 |
| DUF655 |
| Diphthamide_syn |
| FbpA |
| HMG-CoA_red |
| Ham1p_like |
| NDK |
| PPS_PS |
| PTH2 |
| Prefoldin |
| PyrI |
| RNA_pol_L_2 |
| RNA_pol_N |
| RNA_pol_Rpb4 |
| RNA_pol_Rpb6 |
| RNase_HII |
| Rib_5-P_isom_A |
| Ribosom_S12_S23 |
| Ribosomal_L1 |
| Ribosomal_L13 |
| Ribosomal_L14 |
| Ribosomal_L15e |
| Ribosomal_L16 |
| Ribosomal_L21e |
| Ribosomal_L22 |
| Ribosomal_L23 |
| Ribosomal_L26 |
| Ribosomal_L29 |
| Ribosomal_L3 |
| Ribosomal_L31e |
| Ribosomal_L32e |
| Ribosomal_L37ae |
| Ribosomal_L39 |
| Ribosomal_L4 |
| Ribosomal_L44 |
| Ribosomal_L5e |
| Ribosomal_L6 |
| Ribosomal_S11 |
| Ribosomal_S13 |
| Ribosomal_S15 |
| Ribosomal_S17 |
| Ribosomal_S17e |
| Ribosomal_S19 |
| Ribosomal_S19e |
| Ribosomal_S2 |
| Ribosomal_S24e |
| Ribosomal_S27e |
| Ribosomal_S28e |
| Ribosomal_S3Ae |
| Ribosomal_S7 |
| Ribosomal_S8 |
| Ribosomal_S8e |
| Ribosomal_S9 |
| RtcB |
| SecY |
| Spt4 |
| TIM |
| Trm56 |
| TruD |
| V_ATPase_I |
| dsDNA_bind |
| eIF-6 |
| tRNA-synt_1c |
| tRNA-synt_1d |
| tRNA-synt_His |
| vATP-synt_AC39 |
| vATP-synt_E |
