# Supplementary material for: Two Archaeal Metagenome-Assembled Genomes from El Tatio Provide New Insights into the Crenarchaeota Phylum
Source: Genes (Basel). 2021 Mar 9;12(3):391. doi: 10.3390/genes12030391 (PMC7999037; doi:10.3390/genes12030391)
Supplement: Supplementary file 1 [file genes-12-00391-s001.zip › Supplementary/Table S3.docx]

Supplementary table 3. Metabolisms detected for MAG 9-5TAT which were not present in *Thermoproteus uzoniensis.*

| Kegg_module | module_name | module_category | module_subcategory |
| --- | --- | --- | --- |
| M00530 | Dissimilatory nitrate reduction, nitrate => ammonia | Energy metabolism | Nitrogen metabolism |
| M00804 | Complete nitrification, comammox, ammonia => nitrite => nitrate | Energy metabolism | Nitrogen metabolism |
| M00076 | Dermatan sulfate degradation | Glycan metabolism | Glycosaminoglycan metabolism |
| M00077 | Chondroitin sulfate degradation | Glycan metabolism | Glycosaminoglycan metabolism |
| M00924 | Cobalamin biosynthesis, anaerobic, uroporphyrinogen III => sirohydrochlorin => cobyrinate a,c-diamide | Metabolism of cofactors and vitamins | Cofactor and vitamin metabolism |
| M00925 | Cobalamin biosynthesis, aerobic, uroporphyrinogen III => precorrin 2 => cobyrinate a,c-diamide | Metabolism of cofactors and vitamins | Cofactor and vitamin metabolism |
