# Supplementary material for: Two Archaeal Metagenome-Assembled Genomes from El Tatio Provide New Insights into the Crenarchaeota Phylum
Source: Genes (Basel). 2021 Mar 9;12(3):391. doi: 10.3390/genes12030391 (PMC7999037; doi:10.3390/genes12030391)
Supplement: Supplementary file 1 [file genes-12-00391-s001.zip › Supplementary/Table S2.docx]

Supplementary table 2. Metabolisms detected for MAG 47-5TAT which were not present in *Sulfolobus tokodaii*

| M00631 | D-Galacturonate degradation (bacteria), D-galacturonate => pyruvate + D-glyceraldehyde 3P | Carbohydrate metabolism | Other carbohydrate metabolism |
| --- | --- | --- | --- |
| M00061 | D-Glucuronate degradation, D-glucuronate => pyruvate + D-glyceraldehyde 3P | Carbohydrate metabolism | Other carbohydrate metabolism |
| M00129 | Ascorbate biosynthesis, animals, glucose-1P => ascorbate | Carbohydrate metabolism | Other carbohydrate metabolism |
| M00550 | Ascorbate degradation, ascorbate => D-xylulose-5P | Carbohydrate metabolism | Other carbohydrate metabolism |
| M00855 | Glycogen degradation, glycogen => glucose-6P | Carbohydrate metabolism | Other carbohydrate metabolism |
| M00554 | Nucleotide sugar biosynthesis, galactose => UDP-galactose | Carbohydrate metabolism | Other carbohydrate metabolism |
| M00167 | Reductive pentose phosphate cycle, glyceraldehyde-3P => ribulose-5P | Energy metabolism | Carbon fixation |
| M00169 | CAM (Crassulacean acid metabolism), light | Energy metabolism | Carbon fixation |
