# Supplementary material for: Two Archaeal Metagenome-Assembled Genomes from El Tatio Provide New Insights into the Crenarchaeota Phylum
Source: Genes (Basel). 2021 Mar 9;12(3):391. doi: 10.3390/genes12030391 (PMC7999037; doi:10.3390/genes12030391)
Supplement: Supplementary file 1 [file genes-12-00391-s001.zip › Supplementary/Table S1.docx]

Supplementary table 1. Crenarchaeota genomes used for phylogenetic placement

| NCBI accession number | Crenarchaeota strains |
| --- | --- |
| NC_009954.1 | Caldivirga maquilingensis IC-167 complete genome |
| NC_010525.1 | Pyrobaculum neutrophilum V24Sta complete sequence |
| NC_012726.1 | Sulfolobus islandicus M.16.4 complete sequence |
| NC_014160.1 | Thermosphaera aggregans DSM 11486 complete sequence |
| NC_014205.1 | Staphylothermus hellenicus DSM 12710 complete sequence |
| NC_014374.1 | Acidilobus saccharovorans 345-15 complete sequence |
| NC_014537.1 | Vulcanisaeta distributa DSM 14429 complete sequence |
| NC_014961.1 | Desulfurococcus mucosus DSM 2162 complete sequence |
| NC_015151.1 | Vulcanisaeta moutnovskia 768-28 complete sequence |
| NC_015315.1 | Thermoproteus uzoniensis 768-20 complete sequence |
| NC_003364.1 | Pyrobaculum aerophilum str. IM2 complete sequence |
| NC_015435.1 | Metallosphaera cuprina Ar-4 complete sequence |
| NC_015518.1 | Acidianus hospitalis W1 complete sequence |
| NC_015931.1 | Pyrolobus fumarii 1A complete sequence |
| NC_018001.1 | Desulfurococcus amylolyticus DSM 16532 complete sequence |
| NC_016645.1 | Pyrobaculum ferrireducens complete sequence |
| NC_016070.1 | Thermoproteus tenax Kra 1 complete genome |
| NC_017461.1 | Fervidicoccus fontis Kam940 complete sequence |
| NC_017954.1 | Thermogladius calderae 1633 complete sequence |
| NC_019791.1 | Caldisphaera lagunensis DSM 15908 complete sequence |
| NC_022093.1 | Thermofilum adornatum complete sequence |
| NC_000854.2 | Aeropyrum pernix K1 complete genome |
| NC_022521.1 | Aeropyrum camini SY1 = JCM 12091 complete genome |
| NC_003106.2 | Sulfolobus tokodaii str. 7 DNA complete genome |
| NC_008818.1 | Hyperthermus butylicus DSM 5456 complete genome |
| NC_008701.1 | Pyrobaculum islandicum DSM 4184 complete sequence |
| NC_008698.1 | Thermofilum pendens Hrk 5 complete sequence |
| NC_009033.1 | Staphylothermus marinus F1 complete sequence |
| NC_009376.1 | Pyrobaculum arsenaticum DSM 13514 complete sequence |
| NC_009776.1 | Ignicoccus hospitalis KIN4/I complete sequence |
| NZ_CP009961.1 | Thermofilum uzonense strain 1807-2 chromosome complete genome |
| NZ_CP012172.1 | Metallosphaera sedula strain ARS50-1 chromosome complete genome |
| NZ_CP006867.1 | Ignicoccus islandicus DSM 13165 chromosome complete genome |
| NZ_CP020477.1 | Acidianus manzaensis strain YN-25 chromosome complete genome |
| NZ_CP020364.1 | Sulfolobus acidocaldarius strain DG1 chromosome complete genome |
| NZ_CP029287.2 | Metallosphaera hakonensis JCM 8857 = DSM 7519 strain HO1-1 chromosome complete genome |
| NZ_CP029288.2 | Acidianus sulfidivorans JP7 chromosome complete genome |
| NZ_CP029289.2 | Acidianus brierleyi strain DSM 1651 chromosome complete genome |
| NZ_CP033238.1 | Saccharolobus solfataricus strain SULM chromosome complete genome |
| NZ_AP018553.1 | Sulfodiicoccus acidiphilus strain HS-1 |
| NZ_CP031156.1 | Metallosphaera prunae strain Ron 12/II chromosome complete genome |
| NZ_AP018930.1 | Sulfuracidifex tepidarius strain IC-007 |
| NZ_CP045482.1 | Acidianus ambivalens strain LEI 10 chromosome complete genome |
| NZ_CP045483.1 | Stygiolobus azoricus strain FC6 chromosome complete genome |
| NZ_CP045484.1 | Sulfurisphaera ohwakuensis strain TA-1 chromosome complete genome |
| NZ_CP049074.1 | Metallosphaera tengchongensis strain Ric-A chromosome complete genome |
