# Supplementary material for: Two Archaeal Metagenome-Assembled Genomes from El Tatio Provide New Insights into the Crenarchaeota Phylum
Source: Genes (Basel). 2021 Mar 9;12(3):391. doi: 10.3390/genes12030391 (PMC7999037; doi:10.3390/genes12030391)
Supplement: Supplementary file 1 [file genes-12-00391-s001.zip › Supplementary/Figure S1.docx]

Supplementary figure 1. Sampling site in El Tatio geysers field. Map was taken using Google Maps (<https://www.google.cl/maps>) and sampling site is highlighted in red.
